# Impact of Micro- and Nanoplastics on Mitochondria

**DOI:** 10.3390/metabo12100897

**Published:** 2022-09-23

**Authors:** Seung Eun Lee, Yoojung Yi, Sangji Moon, Hyunkyung Yoon, Yong Seek Park

**Affiliations:** 1 Department of Microbiology, School of Medicine, Kyung Hee University, #26 Kyungheedae-gil, Dongdaemun-gu, Seoul 02447, Korea; 2 Department of Biomedical Science, Graduate School, Kyung Hee University, Seoul 02447, Korea

**Keywords:** mitochondria, micro- and nanoplastics, environmental contaminants, toxicity

## Abstract

Mitochondria are highly dynamic cellular organelles that perform crucial functions such as respiration, energy production, metabolism, and cell fate decisions. Mitochondrial damage and dysfunction critically lead to the pathogenesis of various diseases including cancer, diabetes, and neurodegenerative and cardiovascular disorders. Mitochondrial damage in response to environmental contaminant exposure and its association with the pathogenesis of diseases has also been reported. Recently, persistent pollutants, such as micro- and nanoplastics, have become growing global environmental threats with potential health risks. In this review, we discuss the impact of micro- and nanoplastics on mitochondria and review current knowledge in this field.

## 1. Introduction

Mitochondria originated in specialized eukaryotic cells that engulfed endosymbiotic prokaryotes approximately two billion years ago [1,2,3]. Mitochondria have been referred to as energy-producing organelles or “power plants” of cells that supply over 90% of adenosine triphosphate (ATP) to support cell metabolism [4]. Mitochondria are also involved in the regulation of redox status, ion homeostasis, cell signaling, and cell growth; thus, they play crucial roles in cell survival and cell death mechanisms [5,6,7]. Because of their central roles in cell survival and death, dysfunctional mitochondria contribute to the pathogenesis and progression of various diseases, such as metabolic diseases, neurological diseases, and cancers [8,9,10,11].

Mitochondria produce most of the cellular energy in the form of ATP through the oxidative phosphorylation of carbohydrates and fatty acids. Metabolites produced by ATP synthesis, including nucleic acids, lipids, and proteins, are used for macromolecule biosynthesis. Mitochondria also provide the majority of reactive oxygen species (ROS), and the bulk of mitochondrial ROS is produced in the electron transport chain (ETC) [12,13,14]. Therefore, dysfunctional mitochondria act as harmful ROS generators, causing oxidative stress and triggering apoptosis and cell damage. Upon stress, mitochondrial damage or loss of mitochondrial membrane integrity results in the release of apoptotic factors and the associated caspase activation [15].

Mitochondria contain their own DNA and a unique genetic code that is maternally inherited and differs from that of nuclear DNA. However, most mitochondrial proteins are generated by nuclear DNA translation [16]. The maintenance of mitochondrial DNA (mtDNA) is essential for preventing abnormal mitochondrial function and plays an important role in the pathogenesis of mitochondria-related disorders [17,18]. Additionally, mtDNA mutations and mitochondrial dysfunction have been associated with several diseases such as metabolic syndrome, cancer, neurodegeneration, and diabetes [19,20].

**Figure 1:**
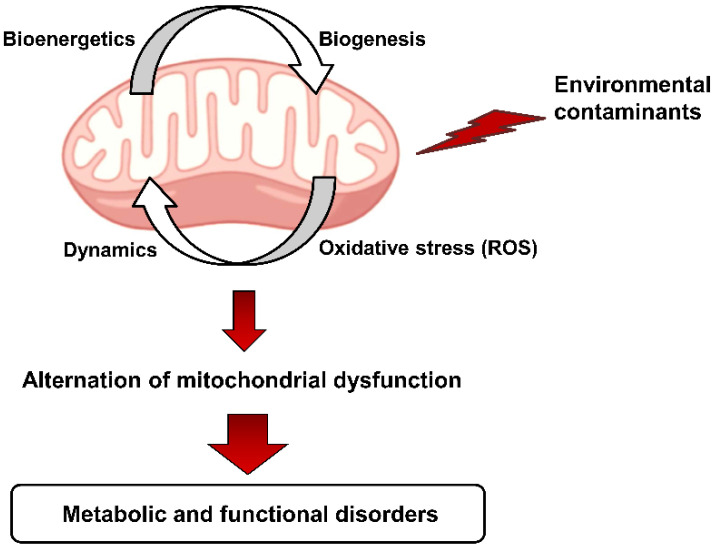
Environmental contaminant-induced mitochondrial dysfunction may lead to various diseases.

Exposure to external stimuli, such as multiple environmental contaminants, affects the normal structure and function of the mitochondria, leading to metabolic and functional diseases [21,22]. Recent studies have provided insights into the involvement of mitochondrial function defects that are induced by the toxicity of environmental contaminants [23,24,25] (Figure 1).

## 2. Environmental Contaminants: Plastics in the Environment

Fossil-based plastics are durable, lightweight, inexpensive, resistant to degradation, and possess thermal and electrical insulation properties [26]. Therefore, global plastic production has increased from 1.5 million tons in the 1950s to approximately 367 million tons in 2020 [27], and this trend is assumed to continue in the coming years. The supply chain of fossil-based plastics, including production, use, management, and waste generation, results in severe environmental problems [28,29]. Among plastic wastes, biodegradable plastics are more susceptible to physical fragmentation (disintegration) than mineralization (degradation); thus, it does not undergo complete biodegradation, which results in smaller sizes of plastic pollutants [30].

### 2.1. Micro- and Nanoplastics

The hazards of micro- and nanoplastics (MNPs) were first discussed in 2004, and their potentially harmful effects on living organisms have recently attracted significant attention [31,32,33,34]. Microplastics (MPs) are artificial polymer particles with sizes less than or equal to 5 mm and are not the end products of plastic waste as they degrade into nanoplastics [35]. Nanoplastics are defined as particles with sizes ranging from 1 nm to 1 μm [36].

**Figure 2:**
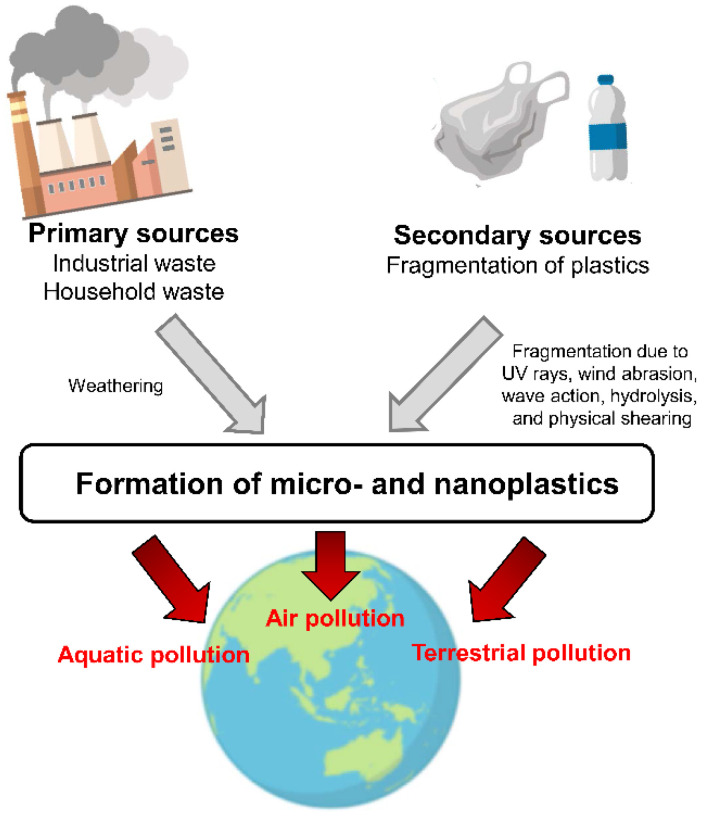
Sources of micro- and nanoplastics in the environment.

MNPs can be classified into two types based on their source of origin: primary MNPs originate from pre-production plastic pellets in manufacturing industries, scrubbers, plastic resin flakes, plastic powder, commercial cleaning abrasives, or fluff used to produce plastic goods [37]. Secondary MNPs originate from the breakdown of larger plastic materials [38], and the frequency of fragmentation depends on the environmental conditions. MNPs penetrate the environment through land-based sources, atmospheric deposition, air transportation, textiles, and aquaculture (Figure 2). In addition, certain degradations, including UV and bacterial, degrade plastics that enter the environment into micro-nanosizes [39]. MNPs comprise the vast majority of plastic contaminants and have become abundant in the global environment [40,41,42].

### 2.2. Environmental Pollution with Microplastics

Globally, over 360 million tons of plastic are produced annually, and it is estimated that more than 8 million tons of plastic are dumped into the oceans annually [43]. The abundance of MPs is approximately 10^3^–10^4^ particles/m^3^ in tidal sediments, 0.1–1 particles/m^3^ in surface waters, and over 10^4^ particles/m^3^ in deep-sea sediments [44]. Rivers are the foremost route for transporting plastic waste from land to sea. The concentration of MPs varies from 0.16 to 3438 particles/m^3^ in North and South American freshwater reservoirs, 0.28 to 1265 particles/m^3^ in European rivers, and 293 to 19,860 particles/m^3^ in Asian water bodies [45].

MPs are ubiquitously found in soil environments such as farmlands, greenhouses, home gardens, and floodplain soils [46]. Sewage irrigation is also among several sources of MPs in soil; in the floodplain soils of Spain, the abundance of MPs was higher in sewage-irrigated agricultural fields (5190 particles/kg) than in non-sewage-irrigated agricultural fields (2030 particles/kg) [47].

Airborne MPs have been detected in Shanghai, Paris, and London [48,49,50]. Atmospheric MPs originate from textiles, whereas non-fibrous particles mostly originate from the decomposition of packaging materials.

### 2.3. Microplastics in the Food Chains and Food

Current food production systems use large amounts of plastic materials for food packaging. Considerable use of plastics has resulted in the penetration of MPs into the food chain, resulting in high exposure to consumers. For example, the abundance of MPs in terrestrial food chains has been reported in chicken stomachs (5.1 particles/g), feces (105 particles/g) [34,51], and sheep feces (1000 particles/g) [52]. Studies have shown the presence of MPs in water, honey, beer, seafood, and sugar [53].

## 3. Toxicities of Micro- and Nanoplastics on Mitochondria

### 3.1. Toxicities in Human Cells

The exposure routes of MNPs to humans include inhalation of plastic-contaminated air or ingestion of plastic-contaminated food and water [54,55]. Thus, the respiratory and digestive systems are the first sites of contact for MNPs. MNPs induce systemic toxicity by penetrating cell membranes and internalizing into cells.

The cytotoxicity of MNPs has been evaluated by analyzing cell viability, intracellular ROS levels, mitochondrial membrane potential levels, and apoptosis in human pulmonary cells [56]. The major mechanism of the toxic effect of MNPs on cells may be the increase in ROS caused by oxidative stress, which in turn leads to a decrease in mitochondrial membrane potential. This study provides information on the toxicity of MNPs at environmental concentrations in human pulmonary cells, which helps to improve the risk cognition of MNPs in the respiratory system.

MNPs cause NADPH oxidase 4 (NOX4)-mediated mitochondrial dysfunction, as demonstrated by membrane potential changes and impaired cellular energy metabolism in the respiratory epithelium [57]. This suggests that MNPs induce epithelial-to-mesenchymal transition in human lung adenocarcinoma A549 cells via multiple mechanisms and that NOX4 is a vital mediator in this process. These findings contribute to our understanding of the toxicological mechanisms of MNPs in the respiratory system.

Lin et al. identified that human liver and lung cells exposed to MNPs experienced mitochondrial damage, as evidenced by the overgeneration of mitochondrial ROS, alterations in the mitochondrial membrane potential, and repression of mitochondrial respiration [58]. This study revealed the MNP-evoked mitochondrial dysfunction and metabolic toxicity pathways in target human cells, providing new insights into the possibility of adverse outcomes in human health.

A recent study suggested that MNP exposure directly augments mitochondrial damage and dysfunction and that mitochondrial breakdown results in mtDNA release into the cytoplasm. Accumulation of MNPs in hepatocytes was detected in vivo and in vitro. MNPs lead to nuclear DNA and mtDNA damage, and subsequent activation of the cGAS/STING signaling pathway was involved in mediating liver fibrosis. This study provides valuable insights into the potential risks and mechanisms of MNP-induced hepatic toxicity and fibrosis [59].

Wu et al. investigated whether exposure to MNPs alters mitochondrial depolarization and ATP synthesis, thereby inhibiting ATP-binding cassette transporter activity and enhancing toxicity in Caco-2 cells [60]. This study provides basic information on the toxicity of MNPs in human intestinal cells, which is useful for assessing the risk posed by MNPs in humans. Similarly, Wang et al. provided evidence that uptake of the MNPs was related to increased cellular oxidative stress and mitochondrial depolarization [61].

A recent study suggested that MNPs may cause changes in mitochondrial ROS, BCL2-associated agonist of cell death proteins, endoplasmic reticulum stress-related proteins, inflammation-related proteins, and autophagy-related proteins, leading to kidney damage and protein leakage [62]. These results suggest that exposure to MNPs may be a risk factor for poor kidney health.

Florance et al. revealed that MNP-enhanced lipid accumulation is accompanied by mitochondrial oxidative stress and loss of mitochondrial membrane potential, which is presumably restored in human macrophages [63]. This study demonstrated that MNPs induce lipid accumulation in macrophages accompanied by acute oxidative stress and induce macrophage foam cell formation, a characteristic feature observed in the pathology of atherosclerosis.

MNPs cause endothelial cell dysfunction by diminishing mitochondrial function and increasing pro-inflammatory cytokines, thereby triggering apoptosis [64]. This study also revealed differences in gene expression and metabolite levels between MNP-treated endothelial cells.

MNPs augment neurotoxicity in SHSY-5Y cells through the activation of autophagy and mitochondrial dysfunction, which are modulated by mitochondrial oxidative stress. Hence, mitochondrial damage caused by oxidative stress may be involved in the pathological mechanisms of MNP-evoked neurodegenerative diseases [65].

Salimi et al. revealed that MNPs are toxic to human lymphocytes, owing to excessive ROS formation, lysosomal/mitochondrial damage, lipid peroxidation, and glutathione depletion, ultimately resulting in cytotoxicity [66]. This study showed that human lymphocytes are more sensitive to MNP toxicity than fish lymphocytes.

### 3.2. Toxicities in Other Animal Cells

Liu et al. demonstrated the mitochondrial toxicity of MNPs, thus providing a basis for understanding the causes of sperm damage caused by MNPs. MNPs can damage the mitochondrial structure of GC-2 cells, a mouse spermatocyte line, decrease ATP content, diminish membrane potential, and destroy the integrity of the mitochondrial genome, leading to an imbalance in mitochondrial dynamic homeostasis, which induces mitochondrial autophagy. This study explored the effects of MNPs on the mitochondria of germ cell lines, providing support for further research on the effects of MNPs on reproductive health [67].

The exposure of rat basophilic leukemia RBL-2H3 cells to MNPs resulted in severe mitochondrial damage and apoptosis. Eventually, MNPs induce oxidative stress, damage organelles, and trigger apoptosis by augmenting the modulator of apoptosis-1 expression. Consequently, MNP-induced oxidative stress, organelle damage, cell cycle arrest, and apoptosis are correlated events [68].

MNPs can decrease cell viability, induce cell apoptosis, upregulate apoptosis-related protein expression, elicit ROS production, alter mitochondrial membrane potential, and dysregulate mitochondrial function in murine splenic lymphocytes [69].

MNP-exposed macrophages revealed a decrease in mitochondrial membrane potential, which suggests that phagocytosis of MNPs by murine macrophages can induce an immunometabolic active state [70]. These findings suggest a significant impact of alterations in macrophage metabolism caused by MNP exposure on immunity and inflammation.

Zhang et al. demonstrated that MNPs lead to changes in mitochondrial function and glycogen synthase kinase-3β (GSK-3β) and its associated gene expression in mice via the PI3K/AKT pathway, which eventually leads to the apoptosis of neurons [71]. This finding facilitates the understanding of the neurotoxic effects of MNPs on neurons in the cerebra of mice and helps distinguish the important role of maintaining normal mitochondrial function in protecting cerebrum health.

Liang et al. applied single-nucleus RNA sequencing (snRNA-seq) to mouse brains to examine the transcriptional changes at the single-cell level. Furthermore, to confirm the snRNA-seq findings, the misfolded protein levels, inflammation levels, and ATP content in different regions of the mouse brain were evaluated. MNPs may primarily induce mitochondrial dysfunction and energy metabolism disorders in neurons, particularly in excitatory neurons. Additionally, MNPs may cause disorders of ATP metabolism and mitochondrial and synaptic function regulation in astrocytes and may be involved in neurodegeneration [72].

A recent study revealed that MNPs might suppress antioxidative reactions and induce oxidative stress, leading to mitochondrial damage and cell death in ionocytes, ultimately impairing skin functions such as ion uptake, pH regulation, and ammonia excretion in zebrafish embryos [73]. This study expands our knowledge of the potential toxicity of MNPs in aquatic animals.

MNPs can induce neurodevelopmental toxicity depending on particle size, which is mediated by mitochondrial damage and dopamine reduction. Data from the mutant test demonstrated that improved expression of sel-12 and hop-1 is involved in the regulation of MNP-induced oxidative stress, mitochondrial damage, and neurodevelopmental toxicity in *Caenorhabditis elegans* [74].

Recently, MNPs, which have emerged as a serious issue in the field of environment and food safety, have been known to induce cell damage and affect cell survival, death, inflammation, and immune responses. Therefore, this review focuses on the association between mitochondria that are closely related to cell growth, survival, and death with various abnormalities induced by MNPs and diseases caused by mitochondrial dysfunction (summarized in Table 1). These findings and their implications should be discussed in the broadest possible contexts, including genomics and epi-genomics, in future research.

## 4. Conclusions

Mitochondria are eukaryotic cellular organelles that play a chief role in cells by metabolizing nutrients and producing the “universal energy currency”, ATP, and are responsible for various processes such as biosynthesis, bioenergetics, and signaling [75,76]. Dysregulated function of mitochondria has been proved to be essential for the pathogenesis and development of various diseases. Mitochondria are the main source of ROS production, redox molecule generation, and calcium storage; hence, mitochondria function as regulators of multiple related signaling pathways that contribute to a range of pathologies [77,78]. Mitochondrial structural and functional modifications have also been reported to be involved in cancer, metabolic syndromes, aging, and other diseases, such as stroke, ischemia, diabetes, obesity, heart disease, and neurodegenerative diseases.

**Figure 3:**
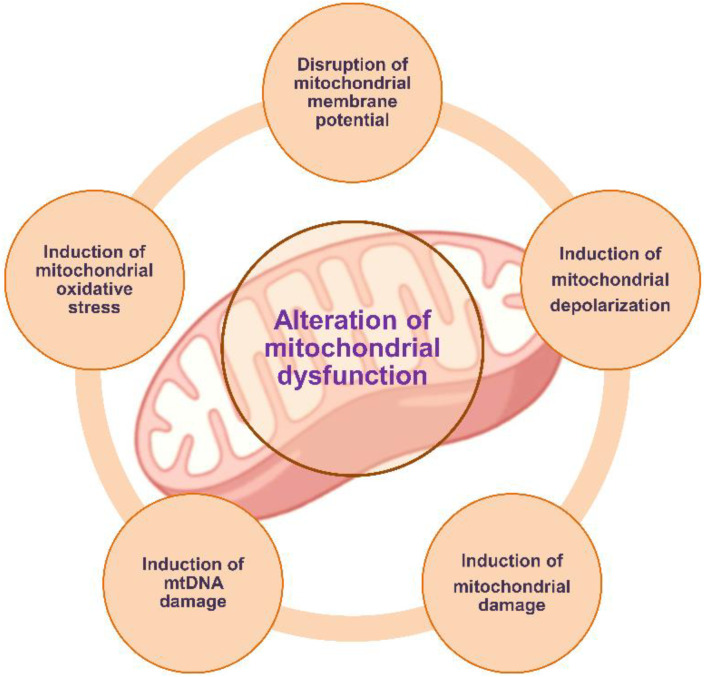
Role of mitochondria in response to micro- and nanoplastics toxicity.

Numerous studies have shown that exposure to environmental contaminants leads to impairment of mitochondrial function and disruption of mitochondrial dynamics (Figure 3). However, mitochondria-dependent mechanisms associated with MNPs exposure are still poorly understood.

This review focuses on understanding the effects of MNPs to the structure and functions of mitochondria and its impact in the pathogenesis of various diseases. A better understanding of the role of mitochondria after exposure to MNPs will provide a broader understanding of cellular systems.

## Figures and Tables

**Table 1:** Summary of micro- and nanoplastic (MNP) toxicity on mitochondria.

Models	Mechanism	References
A549 (human alveolar epithelial cells)	Disruption of mitochondrial membrane potential	Zhang et al., 2022 [56]
	Alteration of mitochondrial dysfunction	Halimu et al., 2022 [57]
L02 (human hepatic cells) and BEAS-2B (human lung epithelial cells)	Alteration of mitochondrial dysfunction (disruption of mitochondrial membrane potential and suppression of mitochondrial respiration)	Lin et al., 2022 [58]
Human liver and mice liver cells	Induction of mtDNA damage	Shen et al., 2022 [59]
Caco-2 (human colon adenocarcinoma cells)	Induction of mitochondrial depolarization	Wu et al., 2019 [60]
	Induction of mitochondrial depolarization	Wang et al., 2020 [61]
HK-2 (human kidney proximal tubular epithelial cells) and in the kidneys of mice	Alteration of mitochondrial dysfunction	Wang et al., 2021 [62]
Human and murine macrophages	Disruption of mitochondrial membrane potential and induction of mitochondrial oxidative stress	Florance et al., 2022 [63]
HUVECs (human umbilical vein endothelial cells)	Alteration of mitochondrial dysfunction	Zhang et al., 2022 [64]
SHSY-5Y (human neuroblastoma cells)	Alteration of mitochondrial dysfunction	Tang et al., 2022 [65]
Human lymphocytes	Induction of mitochondrial damage	Salimi et al., 2022 [66]
GC-2 (mouse spermatocyte cells)	Induction of mitochondrial damage	Liu et al., 2022 [67]
RBL-2H3 (rat basophilic leukemia cells)	Induction of mitochondrial damage	Liu et al., 2022 [68]
Murine splenic lymphocytes	Disruption of mitochondrial membrane potential	Li et al., 2022 [69]
Murine macrophages	Reduction in mitochondrial respiration	Merkley et al., 2022 [70]
NS20Y (mouse neuroblastoma cells)	Alteration of mitochondrial dysfunction	Zhang et al., 2022 [71]
Mouse brain	Alteration of mitochondrial dysfunction	Liang et al., 2022 [72]
Zebrafish embryos	Induction of mitochondrial damage	Kantha et al., 2022 [73]
*Caenorhabditis elegans*	Induction of mitochondrial damage	Liu et al., 2020 [74]

## Data Availability

Not applicable.
